# Prognostic significance of osteopontin expression in gastric cancer: a meta-analysis

**DOI:** 10.18632/oncotarget.11936

**Published:** 2016-09-10

**Authors:** Xiaobin Gu, Xian-Shu Gao, Mingwei Ma, Shangbin Qin, Xin Qi, Xiaoying Li, Shaoqian Sun, Hao Yu, Wen Wang, Dong Zhou

**Affiliations:** ^1^ Department of Radiation Oncology, Peking University First Hospital, Peking University, Beijing, China

**Keywords:** gastric cancer, osteopontin, meta-analysis, biomarker, prognosis

## Abstract

**Background:**

Accumulated studies have exploited the association between osteopontin (OPN) expression and survival of patients with gastric cancer (GC), however, the results were controversial. Thus, we performed a meta-analysis, aiming to investigate the prognostic role of OPN for GC patients and to explore the association between OPN and clinicalpathological features of GC.

**Results:**

A total of ten studies involving 1775 patients were included in final meta-analysis. Of the included studies, nine were conducted on Asian patients and one was performed on Caucasian patients. Regarding OPN detection, immunohistochemistry (IHC) was used on tissue specimens in eight studies and enzyme linked immunosorbent assay (ELISA) was used on plasma specimens in two studies. The pooled data showed that high OPN expression was correlated with poor OS (HR = 1.59, 95% CI: 1.15–2.22, *p* = 0.006). Subgroup analyses demonstrated that OPN had enhanced prognostic value for Asian patients (HR = 1.64, 95% CI = 1.11–2.41, *p* = 0.012) and for patients receiving surgical resection (HR = 1.6, 95% CI = 1.04–2.48, *p* = 0.034). In addition, the results also showed that elevated OPN expression was associated with lymph node metastasis, TNM stage, depth of invasion, tumor size and distant metastasis in GC.

**Methods:**

Relevant studies were retrieved through PubMed, Embase and Web of Science. Combined hazard ratio (HR) and 95% confidence interval (CI) were calculated to assess the association between OPN and overall survival (OS). Subgroup analyses and publication bias were also conducted.

**Conclusions:**

OPN overexpression was correlated with poor OS and clinical features reflecting high aggressiveness in patients with GC. OPN was a promising prognostic biomarker for GC.

## INTRODUCTION

Gastric cancer (GC) is the fourth most commonly diagnosed cancer and the third leading cause of cancer related deaths among all cancer types worldwide [1]. In 2012, there were 951,600 incident cases (6.75% of all new cancer cases) with 723,100 deaths [2]. Although the morbidity and mortality of GC in 2012 were both slightly declined compared with those in 2008 [1], the prognosis of GC was still poor. The 5-year overall survival (OS) rate of GC is about 20% in most parts of the world [3], and prognosis is much worse for metastatic or recurrent GC than for localized disease. The median OS for metastatic GC is approximately one year, even when patients are treated with chemotherapy [4]. In the past several decades, various prognostic factors including *H. pylori* infection, family history, tumor stage and salt intake have been identified and been applied to aid therapeutic regimens selection, however, these parameters still lack accuracy for prediction [5]. Therefore, it is urgently needed to find novel prognostic biomarkers to provide sufficient and precise information for clinical outcomes estimation in GC.

Osteopontin (OPN) is a secreted phosphorylated glycoprotein component of the extracellular matrix (ECM). OPN mediates a variety of physiological and pathological processes including vascularization, bone resorption and tissue remodeling, atherosclerosis and autoimmune diseases. Meanwhile, OPN as a multifunctional protein has also been involved in every single step of cancer progression including cancer cell adhesion, tumor invasion, metastasis and angiogenesis [6–8]. Moreover, OPN was overexpressed in different solid tumors, including, but not limited to breast cancer [9], non-small cell lung cancer [10], colorectal cancer [11], hepatocellular carcinoma [12] and glioma [13]. Accumulated studies [14–23] also measured the OPN expression in primary tumor tissues or plasma in GC patients and investigated the clinical relevance between OPN and survival outcomes. However, the results of these studies were controversial and inconclusive. Therefore, we collected the most recent and relevant publications and comprehensively explored the association between OPN expression and OS as well as clinicopathological features of GC patients by using meta-analysis.

## RESULTS

### Study selection

**Figure 1:**
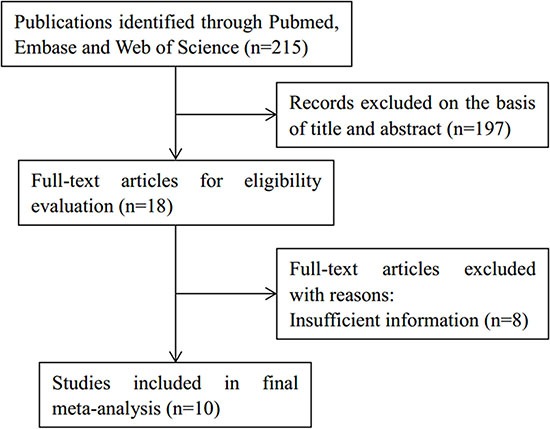
Study selection flow chart

Initial literature search identified 142 relevant records through above-mentioned databases. After title and abstract reading, 125 studies were eliminated. Then, seventeen full-text articles were evaluated for eligibility. Subsequently, seven studies were excluded because they lacked necessary information for meta-analysis. Finally, ten studies [14–23] published from 2007 to 2016 met the criteria for meta-analysis. The results of the search strategy were summarized in Figure 1.

### Characteristics of included studies

The total number of patients was 1775, ranging from 53 to 346 patients per study. The basic characteristics of all included studies are presented in Table 1. Nine of the ten studies were conducted in Asia [14–22] and one study [23] was performed in Italy. Regarding OPN detection, immunohistochemistry (IHC) was used in eight studies [14, 15, 17–21, 23] and enzyme linked immunosorbent assay (ELISA) was used in two studies [16, 22]. Studies using IHC detected OPN expression in primary tumor tissues [14, 15, 17–22] and studies using ELISA measured OPN in plasma[16, 23]. For GC treatment, eight studies [14–21] used surgical resection, one study [22] used chemotherapy and one study [23] exploited surgical resection and chemotherapy. For clinical outcomes analyses, eight studies [14–16, 19–23] investigated both the association between OPN and overall survival (OS) and clinical features, while two studies [17, 18] only exploited the correlation between OPN and clinical factors of GC patients. The NOS scores of included studies were from 7 to 9.

**Table 1:** Characteristics of studies included in the meta-analysis

### OPN expression and overall survival of gastric cancer

**Figure 2:**
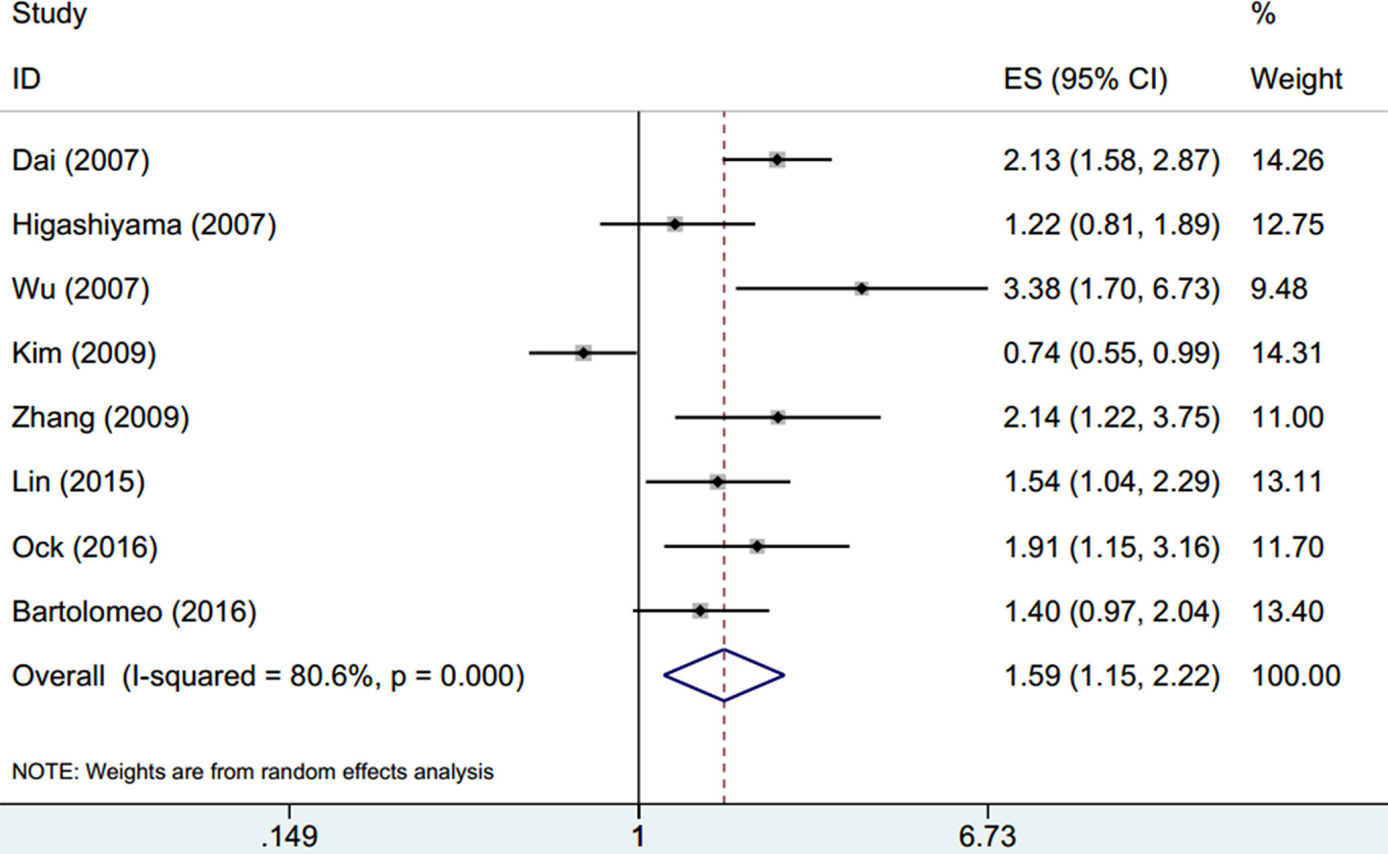
Forest plot of hazard ratio (HR) for overall survival of gastric cancer

There were eight studies [14–16, 19–23] explored the relationship between OPN expression and OS of GC patients. Overall, high OPN expression was associated with poor OS in patients with GC (HR = 1.59, 95% CI: 1.15–2.22, *p* = 0.006; Figure 2), although obvious heterogeneity (I^2^ = 80.6%, *P*_h_ < 0.001) was found. To further investigate the prognosis role of OPN for OS in different subgroups, we conducted subgroup analysis. As shown in Table 2, OPN overexpression had enhanced capability to predicted poor OS in Asian ethnic patients (HR = 1.64, 95% CI = 1.11–2.41, *p* = 0.012), for patients receiving surgical resection (HR = 1.6, 95% CI = 1.04–2.48, *p* = 0.034) and when ELISA was used to measure OPN (HR = 2.33, 95% CI = 1.55–3.51, *p* < 0.001). However, the results demonstrated that OPN expression had no prognostic significance for patients with Caucasian ethnic background (HR = 1.4, 95% CI = 0.87– 2.03, *p* = 0.076), patients receiving chemotherapy or surgery and chemotherapy treatment or when IHC was used to detect OPN. Notably, as for detection methods, OPN remained a prognostic marker by ELISA method (HR = 2.33, 95% CI = 1.55–3.51, *p* < 0.001), and OPN was also associated with poor OS when tested by IHC, although it did not have statistical significance (*p* = 0.06).

**Table 2:** Association between OPN and overall survival of GC patients in meta-analysis

Factors	Studies (*n*)	Effects model	HR (95%CI)	*p*	Heterogeneity	
					I^2^(%)	P_h_
Overall survival	8	Random	1.59 (1.15–2.22)	0.006	80.6	< 0.001
Ethnicity						
Asian	7	Random	1.64 (1.11–2.41)	0.012	83.3	< 0.001
Caucasian	1	-	1.4 (0.97–2.03)	0.076	-	-
Treatment						
Surgery	6	Random	1.6 (1.04–2.48)	0.034	85.6	< 0.001
Chemotherapy	1	-	1.91 (1.15–3.17)	0.012	-	-
Surgery+chemotherapy	1	-	1.4 (0.97–2.03)	0.076	-	-
Detection method						
IHC	6	Random	1.42 (0.99–2.04)	0.06	82.3	< 0.001
ELISA	2	Fixed	2.33 (1.55–3.51)	< 0.001	41.8	0.19
Study design						
Restrospective	6	Random	1.49 (1.01–2.2)	0.046	83.3	< 0.001
Prospective	2	Random	2.07 (0.88–4.88)	0.097	79.5	0.027

### OPN expression and clinicopathological features of gastric cancer

To further identify the impact of OPN on clinicopathological features of GC, we investigated the correlation between OPN overexpression with ten variables including age, gender, lymph node metastasis, TNM stage, depth of invasion, tumor size, distant metastasis, tumor location, lymphatic invasion and venous invasion. The combined data showed that elevated OPN expression was associated with lymph node metastasis (OR = 2.03, 95% CI = 1.38–2.98, *p* < 0.001), TNM stage (OR = 1.83, 95% CI = 1.22–2.75, *p* = 0.004), depth of invasion (OR = 1.97, 95% CI = 1.22–3.17, *p* = 0.005), tumor size (OR = 1.43, 95% CI = 1.05–1.93, *p* = 0.022) and distant metastasis (OR = 2.66, 95% CI = 1.24–5.71, *p* = 0.012) (Table 3). However, the results suggested that there was no significant association between OPN and other clinical factors, including gender, age, tumor location, lymphatic invasion and venous invasion.

**Table 3:** Association between OPN expression and clinical characteristics of GC

Features	Studies (*n*)	Effects model	OR (95%CI)	*p*	Heterogeneity		Publication bias	
					I^2^(%)	P_h_	Begg's p	Egger's p
Gender (male vs. female)	7	Fixed	1.11 (0.88–1.41)	0.378	42.5	0.107	1	0.399
Lymph node metastasis (yes vs. no)	7	Random	2.03 (1.38–2.98)	< 0.001	54.6	0.04	0.881	0.415
TNM stage (III–IV vs. I–II)	6	Random	1.83 (1.22–2.75)	0.004	51.5	0.067	0.851	0.504
Depth of invasion (T3–4 vs. T1–2)	6	Random	1.97 (1.22–3.17)	0.005	57.9	0.037	1	0.664
Age (≥ 60 vs. < 60)	5	Fixed	1.27 (0.95–1.69)	0.102	43.7	0.13	0.806	0.825
Tumor size (≥ 5 cm vs. < 5 cm)	4	Fixed	1.43 (1.05–1.93)	0.022	31.2	0.225	1	0.918
Distant metastasis (yes vs. no)	4	Random	2.66 (1.24–5.71)	0.012	55.6	0.08	0.308	0.193
Tumor location (antrum vs. cardia/fundus)	3	Fixed	1.03 (0.72–1.45)	0.887	29.1	0.244	1	0.891
Lymphatic invasion (yes vs. no)	3	Random	2.39 (0.66–8.69)	0.184	87.2	< 0.001	0.296	0.256
Venous invasion (yes vs. no)	3	Random	1.04 (0.24–4.56)	0.962	87.9	< 0.001	1	0.959

### Publication bias

**Figure 3:**
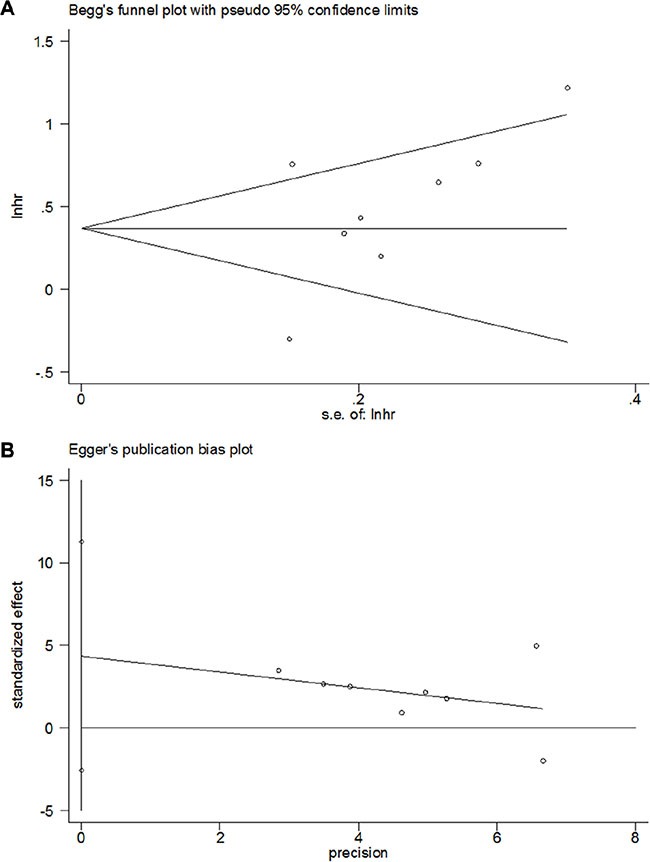
Publication bias for the prognostic value of OPN expression in gastric cancer by (A) Begg's test (*p* = 0.174) and (B) Egger's test (*p* = 0.176)

In the present meta-analysis, using Begg's test and Egger's test, no significant publication bias was detected for the analysis between OPN and OS (Begg's *p* = 0.174 and Egger's *p* = 0.176; Figure 3). In addition, there was no significant publication bias on the analyses between OPN and clinicopathological features (Table 3). Therefore, no publication bias was found in the present meta-analysis, indicating the credibility of our study.

## DISCUSSION

The involvement of OPN in carcinogenesis has been reported in the past decades, especially in promotion of tumor occurrence and metastasis [24]. More recently, it was reported that tumor-derived OPN induced mesenchymal stem cells (MSCs) to cancer-associated fibroblasts (CAFs) transformation to facilitate tumor progression and metastasis in tumor microenvironment [25]. Owing to its pivotal function in pathophysiology of cancer, OPN has been suggested as a novel and promising biomarker for cancer prognosis as well as a therapeutic target in various cancers [26, 27]. However, the prognostic role of OPN for GC was still controversial based on previous reports.

In the present meta-analysis, we included 1775 GC patients from ten studies. The pooled HR and 95% CI suggested that OPN predicted shorter OS in GC, especially for Asian patients and for those who received surgery. Furthermore, through the analyses of association between OPN and clinical features, we found that high OPN expression was more tightly correlated with variables which reflected the aggressiveness and dissemination of this disease including lymph node metastasis, TNM stage, depth of invasion, tumor size and distant metastasis. These results demonstrate that OPN overexpression can be considered and validated as a useful prognostic biomarker and an indicator which represents the invasiveness of gastric cancer. To the best of our knowledge, this is the first meta-analysis to date investigating the prognostic value of OPN in patients with GC.

Previous evidence showed that OPN was a stimulator in tumor progression [26]. Robertson *et al.* showed that OPN regulated the activation of c-Raf-ERK cascade and OPN signaling mediated anti-apoptotic activity [28]. Chakraborty *et al.* demonstrated that OPN could upregulate VEGF expression and promote tumor angiogenesis in breast cancer [29]. In addition, recent studies indicated that OPN also had an immunosuppressive role in tumor milieu. Kim *et al.* reported that tumor-derived OPN could lead to accumulation of peripheral myeloid-derived suppressor cells (MDSC), which were potent immunosuppressive cells [30]. These diverse mechanisms could potentially account for the results that OPN was associated with tumor aggressiveness in this meta-analysis.

We found several previously published meta-analyses also explored the prognostic significance of OPN in different cancers including breast cancer [31], colorectal cancer [32], non-small-cell lung cancer [33] and glioma [34]. Xu *et al.* showed that OPN expression was a predictor for poor overall survival and disease-free survival in breast cancer patients [31]. Zhao *et al.* found that OPN expression was higher in patients with high-grade glioma than patients with low grade glioma [34]. These results from other cancer types were in accordance with findings in our meta-analysis in gastric cancer, which stressed the rationale of recommending OPN as a biomarker for GC patients. Interestingly, we found that OPN was still a significant marker for OS when tested by ELISA, and was almost a significant indicator when used IHC (*p* = 0.06). This result could be caused by the small sample size in IHC detection subgroup, more eligible studies using IHC method could possibly make the pooled results significant.

Although this is the first meta-analysis exploring the prognostic role of OPN for GC, several limitations still need to be stated. First, because we only included full papers in English, selection bias could be possible. Second, heterogeneity was found in the meta-analysis. Although we picked up eligible studies using uniform selection criteria, the difference such study design, patients, OPN detection methods still existed in the included studies, which could be the source of heterogeneity. Third, the sample size was relatively small, because only ten studies were included. Fourth, most patients in this meta-analysis were Asian ethnicities; therefore, the results may be more applicable for Asian populations. As a result, more studies on non-Asian patients are needed.

In summary, the present meta-analysis demonstrated that OPN overexpression was correlated with poor OS in patients with GC and OPN had enhance prognostic value for Asian patients and those underwent surgical resection. Moreover, OPN was associated with clinical features reflecting high aggressiveness of GC. Our results indicated that OPN was a promising prognostic biomarker for GC, which might be more applicable for Asian patients. Due to the limitations of this study, large scale studies with strict study design are still warranted to verify our results.

## MATERIALS AND METHODS

### Search strategy

A thorough literature search was conducted on the electronic platforms of Pubmed, Embase and Web of Science until August 16, 2016. The search strategy included items for ‘OPN’ (e.g. “OPN”, “osteopontin”, “sialoprotein 1”, “secreted phosphoprotein 1”, “bone sialoprotein 1”) and ‘gastric cancer’ (e.g. “stomach neoplasms”(MeSH Terms), “GC”, “gastric carcinoma”, “gastric neoplasm”). Appropriate references of the retrieved studies were also manually reviewed to identify potential inclusions.

### Inclusion and exclusion criteria

Studies meeting the following criteria were included: (1) patients were pathologically diagnosed as gastric cancer; (2) the OPN expression was detected by any approach; (3) study population was classified as high and low OPN expression for analysis; (4) studies investigated the association between OPN expression and survival outcomes or clinical features; (5) the hazard ration(HR) and 95% confidence interval (CI) were directly reported or can be calculated based on the information in the paper ; (6) studies must be published as original articles in English.

Studies were excluded for the following reasons: (1) reviews, letters, comments or meeting abstracts; (2) animal studies; (3) studies lacked sufficient data to extract HRs and 95% CIs.

### Data extraction and quality evaluation

Two investigators (XB Gu and XS Gao) read the eligible studies and extracted data independently. Discrepancies were resolved by discussion. The extracted information included: first author's name, year of publication, patients’ number, country, tumor stage, treatment information, sample source, detection method, cut-off level, follow-up information and HRs with 95% CIs. The qualities of the included studies were assessed by Newcastle-Ottawa Scale (NOS) [35], and studies with ≥ 7 scores were considered as high quality studies.

### Statistical analysis

Pooled HR and 95% CI were calculated to measure the impact of OPN expression on survival of gastric cancer patients. Heterogeneity among studies was evaluated by using a χ^2^-based Cochran Q test and Higgins I^2^ statistic. *P* value for heterogeneity (*P*_h_) < 0.10 or I^2^ > 50% was considered as significant heterogeneity, accordingly, a random-effect model (DerSimonian-Laird method) was used, otherwise, a fixed-effect model (Mantel- Haenszel method) was used. ORs (odds ratios) with 95% CIs were selected to determine the association between OPN expression and clinicopathological variables of GC, such as TNM stage, lymph node metastasis, tumor size and venous invasion. The impact of OPN overexpression on survival or clinicopathological features was regarded as statistically significant if the 95% CI did not overlap with 1. Potential publication bias was examined by using both Begg's test and Egger's test. A *p*-value < 0.05 was considered as statistically significant. All analyses were carried out using STATA 12.0 software (Stata Corporation, Collage Station, Texas, USA).
